# Coordinating Health Care With Artificial Intelligence–Supported Technology for Patients With Atrial Fibrillation: Protocol for a Randomized Controlled Trial

**DOI:** 10.2196/34470

**Published:** 2022-04-13

**Authors:** Liliana Laranjo, Tim Shaw, Ritu Trivedi, Stuart Thomas, Emma Charlston, Harry Klimis, Aravinda Thiagalingam, Saurabh Kumar, Timothy C Tan, Tu N Nguyen, Simone Marschner, Clara Chow

**Affiliations:** 1 Westmead Applied Research Centre University of Sydney Sydney Australia; 2 Blacktown Mount Druitt Hospital Sydney Australia

**Keywords:** atrial fibrillation, interactive voice response, artificial intelligence, conversational agent, mobile phone

## Abstract

**Background:**

Atrial fibrillation (AF) is an increasingly common chronic health condition for which integrated care that is multidisciplinary and patient-centric is recommended yet challenging to implement.

**Objective:**

The aim of Coordinating Health Care With Artificial Intelligence–Supported Technology in AF is to evaluate the feasibility and potential efficacy of a digital intervention (*AF-Support*) comprising preprogrammed automated telephone calls (artificial intelligence conversational technology), SMS text messages, and emails, as well as an educational website, to support patients with AF in self-managing their condition and coordinate primary and secondary care follow-up.

**Methods:**

Coordinating Health Care With Artificial Intelligence–Supported Technology in AF is a 6-month randomized controlled trial of adult patients with AF (n=385), who will be allocated in a ratio of 4:1 to AF-Support or usual care, with postintervention semistructured interviews. The primary outcome is AF-related quality of life, and the secondary outcomes include cardiovascular risk factors, outcomes, and health care use. The 4:1 allocation design enables a detailed examination of the feasibility, uptake, and process of the implementation of AF-Support. Participants with new or ongoing AF will be recruited from hospitals and specialist-led clinics in Sydney, New South Wales, Australia. AF-Support has been co-designed with clinicians, researchers, information technologists, and patients. Automated telephone calls will occur 7 times, with the first call triggered to commence 24 to 48 hours after enrollment. Calls follow a standard flow but are customized to vary depending on patients’ responses. Calls assess AF symptoms, and participants’ responses will trigger different system responses based on prespecified protocols, including the identification of red flags requiring escalation. Randomization will be performed electronically, and allocation concealment will be ensured. Because of the nature of this trial, only outcome assessors and data analysts will be blinded. For the primary outcome, groups will be compared using an analysis of covariance adjusted for corresponding baseline values. Randomized trial data analysis will be performed according to the intention-to-treat principle, and qualitative data will be thematically analyzed.

**Results:**

Ethics approval was granted by the Western Sydney Local Health District Human Ethics Research Committee, and recruitment started in December 2020. As of December 2021, a total of 103 patients had been recruited.

**Conclusions:**

This study will address the gap in knowledge with respect to the role of postdischarge digital care models for supporting patients with AF.

**Trial Registration:**

Australian New Zealand Clinical Trials Registry ACTRN12621000174886; https://www.australianclinicaltrials.gov.au/anzctr/trial/ACTRN12621000174886

**International Registered Report Identifier (IRRID):**

DERR1-10.2196/34470

## Introduction

### Background

Atrial fibrillation (AF) is a chronic health condition that is increasing in prevalence and is associated with substantial morbidity. In 2010, the number of individuals with AF globally was estimated to be 33.5 million, and this number is expected to double by 2050 [1]. AF places a significant burden on the health care system, particularly because of hospitalizations, with health care costs rising faster than those for any other heart rhythm condition [2]. AF is a substantial contributor to stroke, heart failure, cardiovascular events, dementia, and all-cause mortality [3], and in addition to the morbidity associated with these sequelae, symptomatic AF also contributes significantly to reduced quality of life [4].

Integrated care for AF that is both patient-centric and multidisciplinary is now recommended by several guidelines as the optimal way to manage AF and thereby improve quality of life, patient experience, and health outcomes [5-8]. Despite the push for integrated care, implementation of this model of care in a standardized way is difficult for many centers to achieve. Digital health tools have the potential to facilitate the integration and coordination of care between different clinicians (eg, cardiologist and general practitioner [GP]) and the patient [8], as well as provide standardized pathways of care, support, and education that are customizable and with the added potential for delivery at scale. The inclusion of mobile health (mHealth)—delivery of health care and support through mobile technologies—can enhance health care accessibility. Simultaneously, mHealth can improve the reach of clinical services to provide care to remote and isolated patients above and beyond face-to-face clinical service offerings. mHealth interventions have shown promise in prevention and management of cardiovascular conditions [9-21].

Automated telephone calls are a type of mHealth intervention with wide reach because anyone with a telephone—landline, mobile phone, or smartphone—can potentially receive these types of telephone calls [21-23]. *First-generation* automated telephone calls do not have voice recognition capabilities and include unidirectional calls where the person only listens to a recorded or automated script without the option to interact (eg, a reminder to take prescribed medications) or first-generation interactive voice response (IVR), where the user interacts by using the telephone’s keypad (eg, pressing 1 for *Yes* or entering a number for their systolic blood pressure reading using the keypad). Existing systematic reviews of automated telephone calls have focused predominantly on these first-generation interventions, without voice recognition, showing promising but inconsistent effects in promoting medication adherence, physical activity, immunization, screening, and appointment attendance [22-24].

Recent developments in the field of artificial intelligence (AI) have led to the growing use of conversational AI–driven technologies [25-27]. Conversational technologies simulate human conversation with appropriate responses to dialogue using text or voice. Voice-based conversational technologies include smartphone assistants (eg, Apple’s Siri and Amazon’s Alexa) and AI-driven automated telephone calls, such as more recent IVR systems with voice recognition capabilities. These technologies can analyze verbal speech using voice recognition software and natural language processing, and because they are able to interact with users conversationally, they can potentially promote engagement with health interventions [22,25]. Some potential advantages of the voice recognition software are that it might further enhance the acceptability, engagement, and effectiveness of the IVR system [22,28].

To date, only a few randomized controlled trials have assessed the use of AI-driven automated telephone calls with voice recognition in health care. These studies have shown mixed results in screening interventions and pediatric care [29-31] and improvements in postdischarge care and outcomes of patients with acute coronary syndrome (eg, medication adherence and adverse events) [32]. Interventions had wide variation in voice recognition abilities (ie, from understanding only *yes* or *no* responses to understanding more complex phrases), and studies rarely evaluated patient perspectives and satisfaction with the intervention. So far, in AF, our literature search has identified no AI-driven automated telephone interventions; most digital solutions have focused on screening and basic support in smartphone apps, and the success of such solutions has been inconsistent [10-16,33].

### Objectives

In this study, we will evaluate an intervention —*AF-Support*—designed to support patients with AF, which comprises a series of AI-driven automated telephone calls (IVR with voice recognition ability), emails, SMS text messages, and an educational website. We will conduct a randomized controlled trial to evaluate the efficacy of this intervention in improving the primary outcome of AF-related quality of life compared with usual care. Measures of engagement with the intervention and semistructured interviews with intervention participants will be used to evaluate the feasibility of the intervention.

## Methods

### Study Design

**Figure 1:**
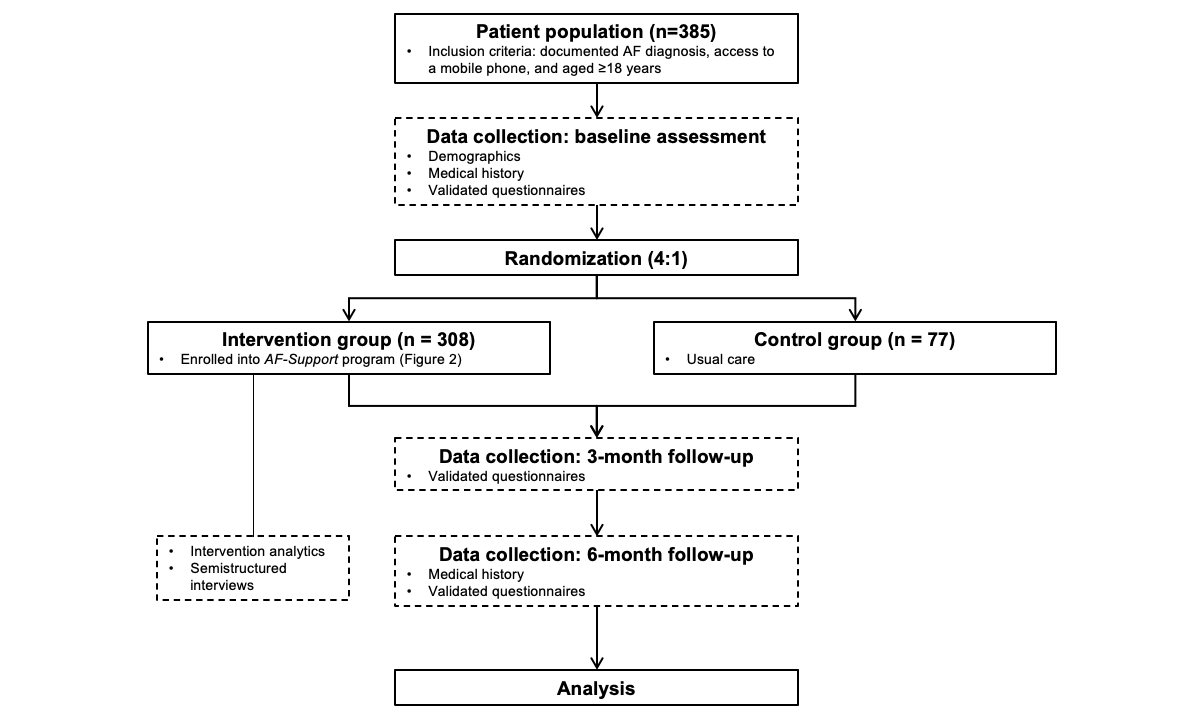
Study flow diagram. AF: atrial fibrillation.

The study will be a 6-month randomized controlled trial (4:1 allocation ratio) among adults with AF to evaluate the efficacy of the intervention *AF-Support* in improving AF-related quality of life compared with a control group receiving usual care. It will be complemented by a mixed methods process evaluation to evaluate feasibility, uptake, and implementation. The 4:1 allocation ratio has been chosen to increase the number of participants exposed to the intervention to enrich data for the assessment of engagement with the intervention, including feasibility, uptake, and acceptability (Figure 1). The trial has been registered in the Australian New Zealand Clinical Trials Registry (trial number: ACTRN12621000174886).

### Study Setting

This study will be conducted in Western Sydney, New South Wales, Australia, with the initial sites proposed to be Westmead Hospital and Blacktown Hospital. Western Sydney serves a culturally, linguistically, and socioeconomically diverse population, with 35% of its population born overseas. Participants will be recruited from cardiology inpatient and outpatient services within the 2 hospitals.

### Participants

Patients will be eligible to participate if they (1) are aged ≥18 years, (2) have a documented diagnosis of AF (including recently diagnosed AF, chronic AF, or paroxysmal or persistent AF), (3) have a mobile phone that is able to receive calls, (4) are able to receive SMS text messages or emails and open weblinks embedded in them, and (5) are competent with the English language as ascertained by the study researchers. Participants will be excluded from the study if they (1) are participating in another AF clinical trial; (2) are pregnant; (3) have a medical illness with anticipated life expectancy of <3 years; (4) are unable to provide written consent; or (5) have a concomitant illness, physical impairment (eg, hearing impairment), or mental condition that in the opinion of the study team or the primary physician could interfere with the conduct of the study, including outcome assessment.

### Randomization and Blinding

The randomization sequence will be generated in R (using the randomizeR package; R Foundation for Statistical Computing) and uploaded to REDCap (Research Electronic Data Capture, Vanderbilt University) software [34]. Allocation concealment will be ensured using management systems in REDCap. We will create separate data access groups to ensure that blinded researchers (eg, outcome assessors and data analysts) will not be able to see randomization lists or access the randomization form in REDCap. A nonblinded researcher will manage randomization of the participants within REDCap as they are recruited. The software will automatically allocate participants to the intervention or control group according to the randomization sequence, ensuring allocation concealment. Randomization will be stratified by center and sex. The allocation sequence will be in a 4:1 ratio (intervention:control). Because of the nature of the study, research study staff involved in recruitment, data collection, and follow-up (eg, semistructured interviews), along with participants and cardiologists, will not be blinded to the randomization outcome.

### Control Group

The control group will receive usual care. Usual care for patients with AF consists of postdischarge instructions from the cardiologist regarding medications, lifestyle modification recommendations, encouragement of follow-up with a GP to be organized by the patient, and additional cardiologist appointments, as needed.

### Intervention: AF-Support Program

#### Overview

**Figure 2:**
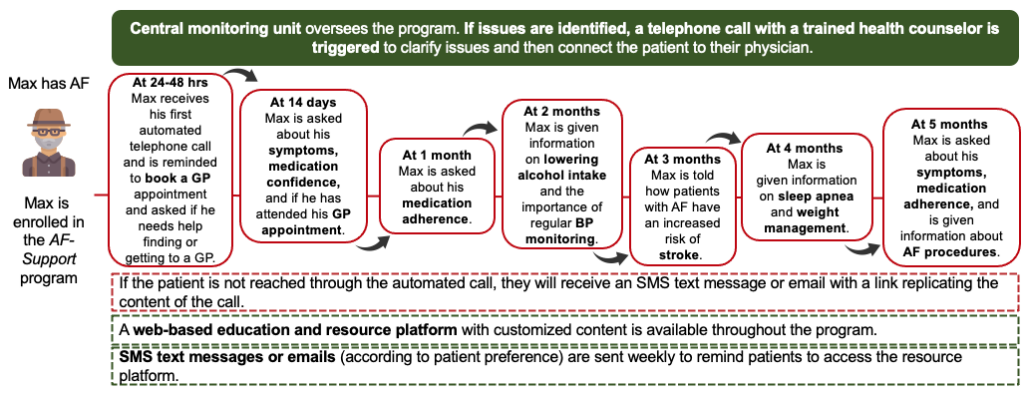
Overview of the AF-Support program. After leaving the hospital or clinic, patients will receive 7 outreaches consisting of automated telephone calls, emails, or SMS text messages, along with access to an educational website. AF: atrial fibrillation; BP: blood pressure; GP: general practitioner.

The AF-Support program comprises 7 patient outreaches (digital *visits*) over 6 months through automated voice calls (IVR with voice recognition) and SMS text messages or emails, supplemented by an educational website (Figure 2). The automated telephone system uses AI to interact with patients and simulate human conversation. The AI underpinning the automated telephone system (ie, conversational AI) [35] includes two main components: automatic speech recognition, which is able to recognize patient voice responses and translate them into text, and natural language processing and understanding, which identifies the semantic and syntactic elements from the user utterance. The system was culturally adapted to Australia and trained to recognize the Australian accent in uttered speech (see *Intervention Development and Patient Involvement* section). In addition, the system has a back-end feature called *Pardon Me*; if the patient’s verbal response is not understood, it will repeat the complete question and ask the patient to press a button corresponding to their response (eg, "please press 1 for always, 2 for often, and 3 for sometimes"), ensuring that the user hears the question more than once and has a chance to respond either verbally or with a number option.

The intervention aims to support patients with AF in self-managing their condition and coordinate primary and secondary care follow-up. The AF-Support program provides education and information on how best to navigate AF care, as well as facilitates risk assessment and provides clinician support as needed, triggered by *red flag* responses to the outreaches.

#### Digital Visits

**Figure 3:**
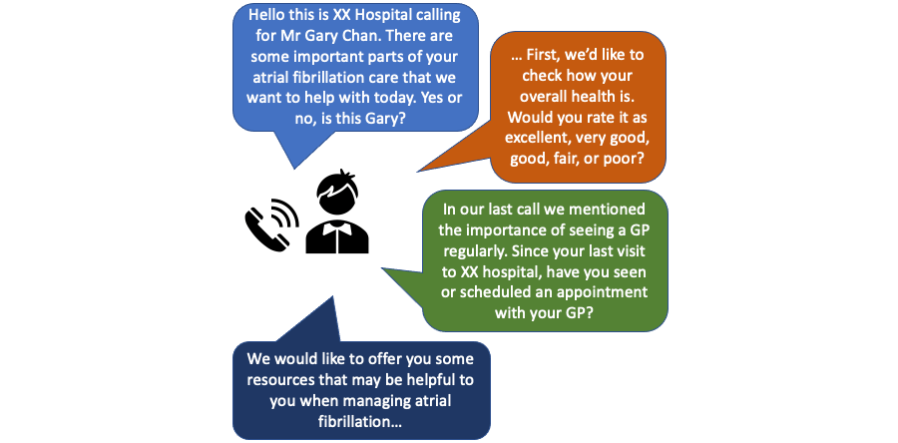
Example of the automated telephone call flow and content. GP: general practitioner.

Outreaches will occur at 24 to 48 hours, 14 days, 1 month, and then monthly until the end of the 6-month program. The automated telephone calls have an average duration of 4 minutes and will start with ensuring that the intended patient has answered the call, with options to call back or reschedule, and assessing their general health. During the automated telephone calls patients will receive AF education and support, as well as verbally respond to risk assessment queries (Figure 3). On the basis of their answers, patients will be presented with information that is tailored to their individual needs. The system is programmed to refer to prior content areas, that is, if a patient has reported in a previous telephone call that they have made a GP appointment, the system will know not to ask again. Each outreach will vary slightly in the information delivered and the risk assessment queries (Table 1).

On the basis of patient responses to risk assessment queries, certain answers will trigger an alert to the central monitoring team and lead to an escalation pathway with clinician support, where needed. Triggering responses include the following: poor overall health status (options ranging from *excellent* to *poor*, where *poor* triggers an alert), impact of AF symptoms on daily life (options ranging from *not at all* to *extremely*, where *extremely* triggers an alert), medication confidence rated 1 or 2 on a scale of 1 to 7 (where 1 is *not confident at all* and 7 is *very confident*), nonadherence to medication (“Are you taking your medications as instructed?”; options are *yes* or *no*), and not having booked or attended a GP appointment within 1 month of hospital discharge. These alerts will be seen and actioned by the central monitoring team and addressed within 24 to 48 hours.

**Figure 4:**
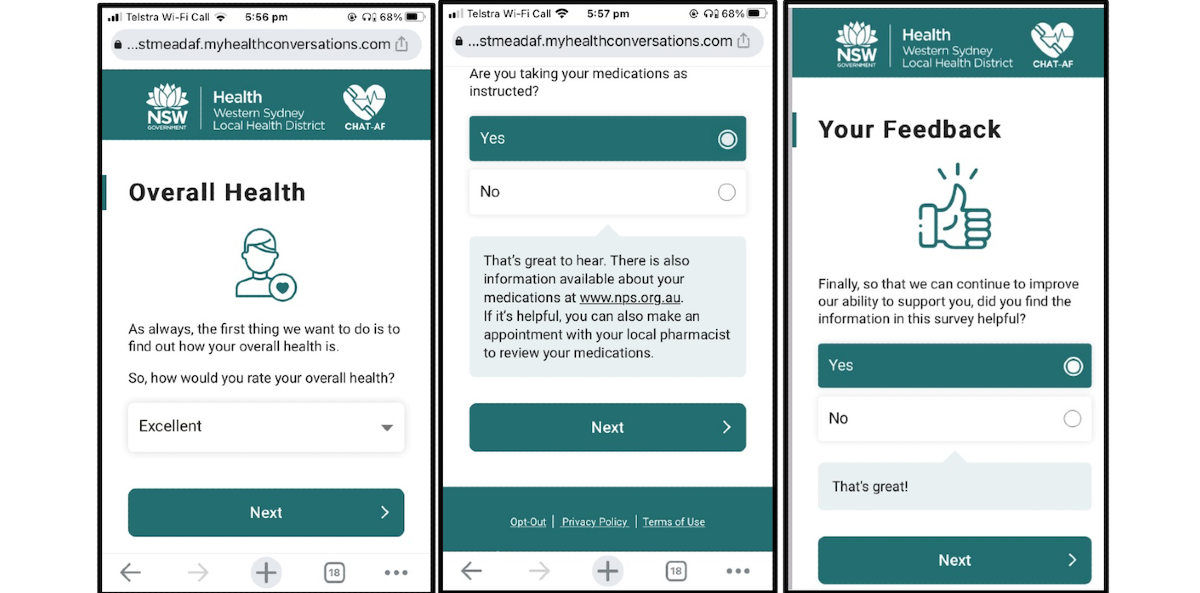
Webpage substitute for the automated telephone call. It replicates content from automated telephone calls, including queries presented in a multiple-choice response format, as seen on a smartphone.

If participants are unable to be reached in the first call attempt, the technology has the capability to leave voicemails directing individuals to a 24/7 telephone number that they can use to complete the call at a more suitable time. If the system is unable to connect with the patient after 3 failed call attempts, an SMS text message or email (depending on patient preference) will be sent containing a link replicating the content of the call, including the option for patients to respond to the queries using a multiple-choice response format (Figure 4).

**Table 1:** Educational information and risk assessment queries delivered in each outreach.

		Outreach number						
		1	2	3	4	5	6	7
**Educational information**								
	General lifestyle information (eg, diet and physical activity)	✓		✓	✓	✓		✓
	Importance of GP^a^ support	✓						✓
	Medication adherence		✓					
	Limiting alcohol intake				✓			
	Blood pressure control				✓	✓		
	Warning signs and risk of stoke					✓		
	Sleep apnea						✓	
	Weight management						✓	
	AF^b^ procedures							✓
**Risk assessment queries**								
	Overall health status	✓	✓	✓	✓	✓	✓	✓
	Regular GP or medical center	✓						
	Transportation barriers to accessing appointments	✓	✓					
	Booked or attended GP appointment		✓	✓		✓		
	AF symptoms and impact on daily life		✓					✓
	Medication confidence and adherence		✓	✓				✓

^a^GP: general practitioner.

^b^AF: atrial fibrillation.

#### Education Website

**Figure 5:**
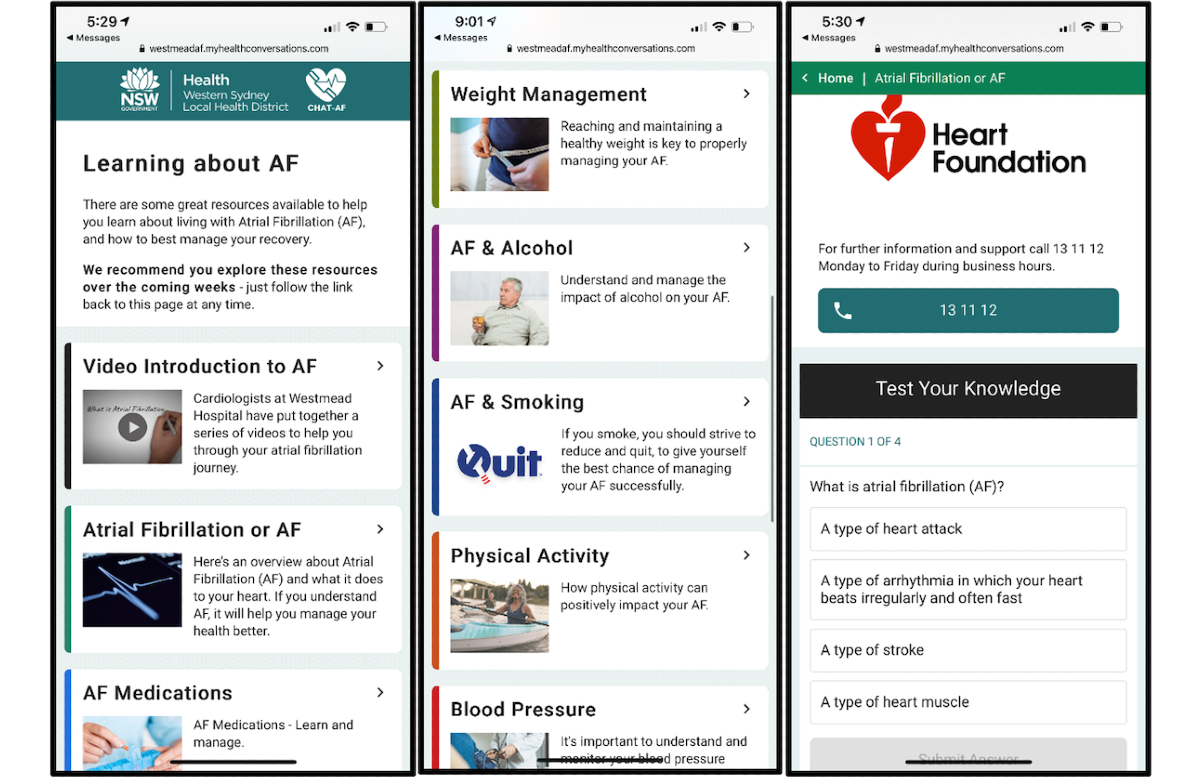
Personalized education website, as seen on a smartphone. It includes information about atrial fibrillation, medications, weight management, alcohol, smoking, physical activity, blood pressure, treatment options (not shown in figure), and other lifestyle information (not shown in figure). The website also has embedded quiz questions to test patients’ knowledge of the educational content. The images contained in these screenshots are stock images; no potentially identifiable patient information is shown.

In addition to these outreaches, an SMS text message or email will be sent to intervention group participants every week to encourage them to visit the educational website (Figure 5). The information on this website will be personalized based on participant information collected at baseline (smoking status, alcohol intake, diagnosis of hypertension, and prescription of anticoagulants or warfarin). The website will consist of various modules, covering general AF information, AF procedures, medications, alcohol, blood pressure, smoking, and weight management, as well as providing general resources. Content will be in the form of embedded videos, text, and images as well as external links to web-based resources, videos, webpages, and brochures from reputable sources (eg, the Heart Foundation and National Prescribing Service). An additional feature of this website is the embedded quiz questions that prompt patients to test their knowledge at the end of each module.

#### Intervention Development and Patient Involvement

The intervention was co-designed with patients, a multidisciplinary group of health care providers, behavioral scientists with experience delivering conversational technology–based health management and wellness programs, and technologists.

The rationale for including automated telephone calls in addition to emails and SMS text messages as part of the intervention was based on 2 recent promising trends to engage patients in health care. First, in the past couple of years, there has been a growing use of conversational AI–driven technologies such as automated telephone calls in health care [25-27], following a similar trend that began in industry and government organizations, with good acceptability and results [36]. These technologies have been applied in different health domains such as mental health support and chronic disease management to enable a conversational interaction with patients and promote engagement with health interventions with the aim of increasing intervention acceptability, use, and effectiveness [22,25-28]. Nevertheless, few randomized controlled trials have assessed their effectiveness so far [29-32], and none in AF.

Second, it has become increasingly common to use multichannel communication to take advantage of different available technologies to interact with users. Having started in marketing and commerce [37], and with growing use in health care [38], multichannel communication aims to improve service quality and user experience by engaging users through several technology channels, depending on preference and the goal of the interaction (eg, bookings, reminders, and education). Therefore, our multidisciplinary team decided to use a combination of automated telephone calls and emails or SMS text messages, according to patient preference, to deliver the intervention. The frequency of the interactions was decided based on the experience of our technology partner in delivering conversational technology–based programs and our own experience in delivering SMS text messaging programs [39-41].

We worked closely with a consumer representative with AF as well as approached patients in cardiology waiting rooms at Westmead Hospital. Patients were approached regardless of whether they had been diagnosed with AF, given that we wanted to ensure that the language in the call scripts was accessible to a lay audience without specific knowledge about AF. Patients were involved in intervention development and refinement, as well as in reviewing the scripts and content of automated calls. Scripts went through several iterations before finalization. Patient feedback was overall positive (eg, “clear and straightforward” and “straightforward and easy to understand”). We received suggestions to use more lay language and avoid the use of medical terminology; for example, the use of *heart specialist* instead of *cardiologist*. Health care providers had input into the content and scripting of the IVR calls, as well as the educational website, to ensure that the information was in accordance with current AF clinical guidelines. We adapted the intervention to incorporate the comments and suggestions received from all stakeholders. The system was culturally adapted to Australia by using a custom voice with an Australian accent and by co-designing the call scripts with Australian patients and health care professionals. The automatic speech recognition engine was trained to recognize different Australian accents in uttered speech. Although the AI models were not specifically trained on a Western Sydney population data set, as part of our process evaluation we aim to assess the adequacy and acceptability of the intervention in this population.

#### Study Outcomes

The primary outcome of the study is AF-related quality of life at 6 months in the intervention group compared with the control group, measured using the AF Effect on Quality-of-Life (AFEQT) questionnaire (total score) [42]. The secondary outcomes include AFEQT domain scores (symptoms, daily activities, treatment concerns, and treatment satisfaction), medication adherence, lifestyle behavioral outcomes, AF knowledge, patient activation, patient care experience, health outcomes, and health care service use. We will also assess the feasibility of the intervention, focusing on acceptability and engagement with different intervention components.

### Data Collection and Study Procedures

#### Overview

A regular screening of upcoming patient appointments and scheduled procedure lists will be conducted by the research team and physicians to identify potential participants. We will approach these participants (1) face-to-face during their visit to obtain consent, (2) over the telephone before they attend their scheduled appointment, (3) or within 48 hours of their visit to the hospital. Eligible individuals who have provided informed consent will be asked to complete a baseline assessment that will consist of study-specific and validated questionnaires. After baseline data collection, patients will be randomized into the study groups. Both control and intervention group participants will complete study-specific surveys and validated questionnaires at baseline, 3 months, and 6 months (Table 2), using a link delivered through SMS text message or email, according to the participants’ preferences. For the purposes of study data collection, SMS text message delivery will be managed by TextCare software (Westmead Applied Research Centre, University of Sydney) [40,41] and email delivery will be managed using REDCap [34]. At 6 months, a purposive sample of participants from the intervention arm will be invited to participate in a semistructured interview. We will also collect patient-specific program analytics throughout the duration of the intervention.

**Table 2:** Data and outcome measures collected at baseline, 3 months, and 6 months.

Outcomes			Data collected		Baseline		3 months		6 months
Primary outcome: AF^a^-related QoL^b^			AFEQT^c^ Questionnaire [42]		✓		✓		✓
**Secondary outcomes**									
	Medication adherence	Self-reported		✓				✓	
	Lifestyle behavioral outcomes	Self-reported		✓				✓	
	AF knowledge	AF knowledge scale [43]		✓				✓	
	Patient activation measure	PAM^d^-13 Questionnaire [44]		✓				✓	
	Patient experience measure	PACIC^e^ Questionnaire [45]		✓				✓	
	Health events	Self-reported and medical records		✓				✓	
	Health care services use	Self-reported and medical records		✓				✓	
	Feasibility (acceptability and engagement)	Semistructured interviews						✓^f^	
	Feasibility (acceptability and engagement)	Program analytics		✓^f^		✓^f^		✓^f^	

^a^AF: atrial fibrillation.

^b^QoL: quality of life.

^c^AFEQT: Atrial Fibrillation Effect on Quality-of-Life.

^d^PAM-13: Patient Activation Measure, 13 items.

^e^PACIC: Patient Assessment of Chronic Illness Care.

^f^Intervention group participants only; program analytics collected throughout the 6 months.

#### Study-Specific Questionnaires

Study-specific questionnaires will collect data on demographics, medication adherence, lifestyle behavioral outcomes, health events, health care service use in past 6 months, and patient medical history (Multimedia Appendices 1-3). Health events, health care service use, and medical history will be collected through study-specific questionnaires (self-reported) and consolidated with hospital medical records.

#### Validated Questionnaires

The primary outcome will be measured using the AFEQT Questionnaire, a reliable and validated 21-item questionnaire measuring self-reported health-related quality of life specific to AF [42]. It consists of scores in four domains (symptoms, daily activities, treatment concerns, and treatment satisfaction), along with a global measure. Scores range from 0 to 100 (higher scores associated with better quality of life). The overall AFEQT score is calculated using a total of the domain scores.

Other validated questionnaires will include the AF Knowledge Scale [43]; the Patient Activation Measure, 13 items (PAM-13) [44]; and the Patient Assessment of Chronic Illness Care (PACIC) Questionnaire [45]. The AF Knowledge Scale [43] will be used to assess patients’ knowledge of AF (1 point is given for each correct response, with the potential total score ranging from 0 to 10; the last item was removed, given that *thrombosis center* is not a term commonly used in Australia). The PAM-13 [44] evaluates patients’ perceived knowledge, skills, and confidence in self-management activities. Question responses are structured according to a Likert scale from 1 (strongly disagree) to 4 (strongly agree), and total scores range from 0 to 100 (higher scores representing more patient activation in disease self-management). To assess patient satisfaction with quality of care, the PACIC Questionnaire [45] will be administered to all study participants at baseline and to intervention group participants only at 6 months. This instrument is a reliable and validated 20-item questionnaire that consists of 5 subscales (patient activation, decision support, goal setting, problem solving, and follow-up) and an overall summary score ranging from 20 to 50 [45].

#### Process Evaluation: Semistructured Interviews and Program Analytics (Intervention Group Only)

A process evaluation will be conducted following the Medical Research Council framework [46] to assess feasibility, engagement, and implementation through the use of semistructured interviews with a sample of intervention participants and program engagement metrics.

At the end of the 6-month program, a sample of participants from the intervention arm will be invited to participate in semistructured interviews. Interviews will explore acceptability and usability, as well as barriers and enablers to engagement; user preferences and patient perspectives regarding specific intervention components (eg, voice calls vs text-based interaction); and impact of the intervention on the patient journey and care integration. Interviews will follow a pilot-tested interview guide (Multimedia Appendix 4). We will use the maximum variation sampling method based on sociodemographic characteristics and engagement metrics to obtain a broad range of views for the semistructured interviews. A minimum of 20 interviews will be conducted; however, additional interviews will be conducted if needed until thematic saturation is reached. Interviews will be conducted through telephone, audio recorded, and manually transcribed for subsequent analysis using NVivo 12 software (QSR International).

Program engagement metrics will be collected and analyzed (eg, number of completed telephone calls, number of queries answered during each telephone call, and number of visits to the educational website). Intervention use metrics will be automatically collected through the IVR system and website analytics. In addition, a log will be kept of responses triggering escalation and subsequent contacts with the research team and clinical resolution.

#### Analysis

A sample size of 385 is required to detect a between-group difference of 7 [47] in the total score (100 points) of the AFEQT Questionnaire [42] with 80% power (*α*=.05; SD 19), accounting for a dropout rate of 10%. All continuous data will be checked for normality before performing parametric tests (2-tailed *t* tests). Appropriate nonparametric tests (eg, Mann-Whitney U test) will be used where data are not normally distributed. Continuous variables will be presented as means and SDs unless they are skewed, in which case medians and IQRs will be used. Categorical variables will be presented as frequencies and percentages. Effect estimates will be reported with 95% CIs. The primary analysis will be by the intention-to-treat principle. In addition, per-protocol analyses will be performed and reported. For the primary outcome (AFEQT total score), groups will be compared using analysis of covariance adjusted for corresponding baseline values. Similarly, other continuous variable secondary outcomes will be adjusted using the corresponding baseline measures (AFEQT domain scores: symptoms, daily activities, treatment concerns, and treatment satisfaction; AF knowledge score; PAM-13 score; PACIC score; BMI; number of cigarettes smoked daily; number of alcoholic drinks consumed per week; exercise minutes per week; daily fruit servings; and daily vegetable servings). For dichotomous outcomes (proportion of self-reported medication adherence; stroke and myocardial infarction rates; proportion of GP, cardiologist, and emergency department visits; and proportion of hospitalizations, catheter ablations, and cardioversions), groups will be compared using a log-binomial regression also adjusting for corresponding baseline values as fixed effect. A statistical analysis plan will be finalized before data lock and unblinding. *P*<.05 will be considered statistically significant. All statistical analyses will be performed using R (version 4.0.2).

Data from interviews will be analyzed using thematic analysis [48] of transcribed audio recordings in NVivo 12 software. Themes will be identified using an inductive data-driven approach, that is, inductive thematic analysis [49]. Inductive thematic analysis is a process of coding the data without trying to fit it into a preexisting coding frame or to analytic preconceptions so that the themes identified are strongly linked to the data [49]. First, we will select relevant information in the data, generating open codes in a codebook (first-cycle coding) [50]. As the analysis progresses, several codes will be added inductively. Second-cycle coding will involve focused coding (ie, to find thematic similarity) and axial coding (ie, to find relationships among the codes) [50]. Identification of themes will occur by sorting the different codes into potential themes and collating all the relevant coded data extracts within the identified themes. Themes will be identified at a semantic level, with analysis starting by organizing data to show patterns in semantic content and then moving to the interpretation of the patterns and their broader meanings and implications [49]. Revisions of the codebook by the authors will be conducted iteratively through comparing and revising codes and emerging themes. After a candidate thematic *map* is reached, the data set will be reread to ensure the quality of the themes and refine them as needed. Reporting will follow the COREQ (Consolidated Criteria for Reporting Qualitative Research) checklist [51].

#### Ethics and Dissemination

Written and informed consent will be obtained from all study participants before commencing any study procedures. Participation in this study will be entirely voluntary, and participants will have the option to withdraw at any point.

The digital technology platform will be hosted on the Amazon Web Service Asia Pacific Region in Sydney, New South Wales, Australia. Data handling and storage will be conducted in accordance with the National Health and Medical Research Council guidelines and Australian Code for Responsible Conduct of Research. Identifiable data collected from this study will be stored on the secure research data store server provided by the University of Sydney and will only be accessible to study researchers. Questionnaire data will be collected electronically and stored on REDCap.

With participant consent, medical history data will be collected from patient medical files for the purpose of this study. As per the Health Records and Information Privacy Act 2002 No 71 (Schedule 1, Section 10 [1a]), the individual to whom the information relates will provide consent for use of their information, in line with the National Health and Medical Research Council’s National Statement.

## Results

Ethics approval was obtained from the Western Sydney Local Health District Human Ethics Research Committee (2020/ETH02546; November 4, 2020). Recruitment started in December 2020, and as of December 2021, a total of 103 patients had been recruited. Results are expected to be published in 2023.

## Discussion

### Anticipated Findings

This study will provide initial data on the efficacy of a novel *AF-Support* program comprising a preprogrammed set of digital visits and conversational AI to provide automated but customized postdischarge AF care designed to support integrated care for patients with AF. It will also provide a detailed evaluation of the implementation, uptake, and overall acceptability of this multichannel digital care intervention. The design of the evaluation has been matched to the goals of the study to both provide new data to enable a better estimate of the efficacy of such a digital care intervention and inform further development of chronic disease digital care interventions. Digital care programs for postdischarge care have the potential to meet the need for increasing management of chronic disease in the community; yet, there is little research on the effectiveness on health outcomes, patient-reported experience, and cost-effectiveness of digital care models.

### Strengths and Limitations of This Study

To the best of our knowledge, this will be the first detailed evaluation of an AI conversational technology to support AF integrated care. The intervention has been co-designed with researchers, multidisciplinary clinicians from tertiary and primary care, informaticians, and patients. The trial is designed with a 4:1 allocation ratio such that most participants will receive the intervention to enable a detailed examination of feasibility and uptake, as well as the implementation process.

The limitations of the study include the 6-month duration and not being powered to assess impact on clinical outcomes such as cardiovascular hospitalizations and events.

### Implications

If successful in this trial, conversational AI interventions may be able to complement clinical care by supporting patients between clinical visits with the GP and cardiac care team. Future software development in this area should explore the integration of such a system with existing electronic medical records in primary and secondary care—a major challenge for the health information technology industry. Enabling seamless clinician access to the data collected by the system (eg, risk assessments such as symptom evaluation) would be an important step toward better integration of AF care. This research will contribute to addressing the gap in knowledge with respect to the role of postdischarge digital care models for supporting patients with chronic disease.

## Appendix

Multimedia Appendix 1 Self-reported study-specific questionnaire 1 (at baseline only).

Multimedia Appendix 2 Self-reported study-specific questionnaire 2 (at baseline and 6 months).

Multimedia Appendix 3 Medical history from hospital records (at baseline and 6 months).

Multimedia Appendix 4 Interview guide.

## Abbreviations

AF: atrial fibrillation
AFEQT: Atrial Fibrillation Effect on Quality-of-Life
AI: artificial intelligence
COREQ: Consolidated Criteria for Reporting Qualitative Research
GP: general practitioner
IVR: interactive voice response
mHealth: mobile health
PACIC: Patient Assessment of Chronic Illness Care
PAM-13: Patient Activation Measure, 13 items
REDCap: Research Electronic Data Capture
