# Supplementary material for: Coordinating Health Care With Artificial Intelligence–Supported Technology for Patients With Atrial Fibrillation: Protocol for a Randomized Controlled Trial
Source: JMIR Res Protoc. 2022 Apr 13;11(4):e34470. doi: 10.2196/34470 (PMC9047758; doi:10.2196/34470)
Supplement: Multimedia Appendix 1 [file resprot_v11i4e34470_app1.docx]

## **Supplement 1: Self-reported study specific questionnaire 1 (at baseline only)**

| **Contact Information** |  |
| --- | --- |
| What is your FIRST name? |  |
| What is your mobile number?  NOTE: Landline numbers are not accepted. |  |
| What is your communication preference? | Text message (SMS)  Email |
| Are you able to access internet and open website on your phone? | Yes  No |
| **Demographics** |  |
| What is your year of birth? |  |
| What is your sex? | Male  Female |
| What ethnic origin best describes you? | Caucasian  Aboriginal / Torres Strait Islander  Chinese  Japanese  Malay  South Asian (Bangladesh, India, Nepal, Pakistan, Sri Lanka)  Other Asian  Arab or Persian  Black African  Coloured African (Sub-Saharan Africa)  Native American Indian  Other |
| What is your highest completed educational qualification? | None  Primary school  Yr 10 School certificate  Yr 12 Higher school certificate  Diploma/Technical  University undergraduate  University postgraduate |
| What is your household income (before tax)? Include pensions (if applicable) | Less than $15,599  $15,600 - $31,199  $31,200 - $51,999  $52,000 - $77,999  $78,000 - $104,000  Over $104,000 |
| **Medical History** |  |
| Have you been diagnosed with heart failure? | Yes  No |
| Do you have hypertension (high blood pressure)? | Yes  No |
| Do you have diabetes? | Yes  No  Type 1  Type 2 |
| Do you have Obstructive Sleep Apnoea (OSA)? | Yes  No |
| Do you have a history of depression? | Yes  No |
| Do you have a history of anxiety | Yes  No |
