# Supplementary material for: Coordinating Health Care With Artificial Intelligence–Supported Technology for Patients With Atrial Fibrillation: Protocol for a Randomized Controlled Trial
Source: JMIR Res Protoc. 2022 Apr 13;11(4):e34470. doi: 10.2196/34470 (PMC9047758; doi:10.2196/34470)
Supplement: Multimedia Appendix 2 [file resprot_v11i4e34470_app2.docx]

## **Supplement 2: Self-reported study specific questionnaire 2 (at baseline and 6 months)**

| **Smoking** |  |
| --- | --- |
| What best describes your smoking status? | Never smoked  Current smoker (if smoked in last 30 days)  Ex-smoker |
| How many cigarettes do you smoke per day? | Cigarettes daily |
| **Alcohol** |  |
| Do you drink alcohol? | Yes  No |
| How many standard drinks of alcohol do you drink on average per week? | Standard drinks per week |
| **Exercise** |  |
| In a typical week, how much time do you spend exercising?  (This includes; brisk walking, swimming, yoga, skipping, cycling, sporting activities) | Exercise minutes per week |
| How many serves of vegetables do you usually eat per day?  (one serve = 1/2 cup cooked vegetables or 1 cup of salad vegetables) |  |
| How many serves of fruit do you usually eat per day?  (One serve = medium piece or two small pieces of fruit  or one cup of diced pieces) |  |
| **AF Medications** |  |
| Are you currently taking anti-coagulation medication? | Yes  No |
| Are you taking Warfarin? | Yes  No |
| **AF Procedures** |  |
| In the past 6 months, have you had a catheter ablation procedure to treat your atrial fibrillation?  Please note a catheter ablation is a procedure that finds where your abnormal heart rhythm comes from and treats that area of the heart. | Yes  No  Unsure |
| In the past 6 months, have you had a cardioversion procedure to treat your atrial fibrillation?  Please note a cardioversion is a procedure that uses an electrical current to reset your heart rhythm. | Yes  No  Unsure |
| Please indicate how many cardioversions you have had in the past 6 months? |  |
| **Health events** |  |
| In the past 6-months have you had a stroke? | Yes  No |
| In the past 6-months have you had a heart attack? | Yes  No |
| **Healthcare Service Utilisation** |  |
| In the past 6 months, how many times have you visited the emergency department (ED) because of possible heart symptoms or problems? |  |
| In the past 6 months, how many times have you been hospitalised because of heart problems (e.g. atrial fibrillation, stroke, heart attack or heart failure)?  Please note this refers to an overnight stay in the  hospital for longer than 12 hours |  |
| In the past 6 months, how many times have you seen a heart specialist (cardiologist)?  Please include today’s appointment and all telephone appointments. |  |
| In the past 6 months, how many times have you seen your GP because of heart problems or heart symptoms?  Please note this includes face-to-face and telephone appointments. |  |
| **Medication Adherence** |  |
| In the last 7 days, on how many days did you miss a dose of any of your prescribed medications? |  |
