# Supplementary material for: Coordinating Health Care With Artificial Intelligence–Supported Technology for Patients With Atrial Fibrillation: Protocol for a Randomized Controlled Trial
Source: JMIR Res Protoc. 2022 Apr 13;11(4):e34470. doi: 10.2196/34470 (PMC9047758; doi:10.2196/34470)
Supplement: Multimedia Appendix 3 [file resprot_v11i4e34470_app3.docx]

## **Supplement 3: Medical History from Hospital Records (at baseline and 6 months)**

| **Anthropometrics** |  |
| --- | --- |
| Height (cm) |  |
| Weight measurement date |  |
| Weight (kg) |  |
| **Blood pressure measurements** |  |
| Blood pressure measurement date |  |
| Systolic Blood pressure (mmHg) |  |
| Dystolic Blood pressure (mmHg) |  |
| **Medical History** |  |
| Heart Failure  Hypertension  Diabetes (excluding gestational diabetes)  Obstructive Sleep Apnoea  History of depression  History of anxiety  Valvular heart disease  Cardiomyopathy  Chronic renal failure  Peripheral vascular (artery) disease  Hyperlipidemia  Coronary artery disease |  |
| **AF-related Medical History** |  |
| Year of diagnosis |  |
| Type of AF (most current) | Paroxysmal  Persistent  Long-term persistent  Permanent |
| Antiarrhythmic Medication | Amiodarone  Atenolol  Bisoprolol  Digoxin  Diltiazem  Carvedilol  Nadalol  Quinidine  Flecanide  Metoprolol  Propanolol  Sotalol  Verapamil  Pindolol  Metoprolol xl  Mexelitine |
| Anticoagulation Medication | Warfarin  Apixaban  Dabiagtran  Clopidogrel  Ticagrelor  Clexane  Rivaroxaban  Aspirin  Prasugrel  Ticlodipine |
| Cardiovascular Medications | Statin  ACEI  ARB  Calcium channel blocker  Neprilysin inhibitor |
| Other Medications |  |
| **AF Procedures** |  |
| Previous catheter ablation in past 6-months | Yes  No |
| Number of cardioversions in past 6 months |  |
| **Health Events** |  |
| Stoke/TIA in past 6 months | Yes  No |
| Myocardial infarction in past 6 months | Yes  No |
