# Supplementary material for: Coordinating Health Care With Artificial Intelligence–Supported Technology for Patients With Atrial Fibrillation: Protocol for a Randomized Controlled Trial
Source: JMIR Res Protoc. 2022 Apr 13;11(4):e34470. doi: 10.2196/34470 (PMC9047758; doi:10.2196/34470)
Supplement: Multimedia Appendix 4 [file resprot_v11i4e34470_app4.docx]

## **Supplement 4: Interview guide**

- What are the parts of having AF that are particularly difficult to live with and manage on a daily basis?
  - Prompt: remembering to take a lot of medications, symptoms, unexpected changes in health, number of consultations with GP and cardiologist
- What are some of things that help you on a daily basis to live with AF or manage your health?
  - Prompt: family support, reminder for taking your medication, appointment reminders, doctor that you’re comfortable with
- Apart from this research study, did you ever use technology to help with your AF or general health?
  - Prompt: googling information about health, using a smartwatch to track your steps, using an app to help with diet or exercise, monitoring ECG
- What are your thoughts about the current intervention you received?
  - Prompt: like/dislike/neutral
- What worked well?
  - Prompts: phone calls and website, text messages or emails (specific parts of the intervention components that they liked)
- What did not work as well?
  - Prompts: phone calls and website, text messages or emails (specific parts of the intervention components that they disliked)
- Do you think that being on this study helped with your overall health?
  - What specifically did you think helped with your overall health, was it the phone calls, the website…?
  - Prompt: quality of life, lifestyle change, knowledge about AF, taking your medication, symptoms
- Do you think that being on this study helped with your healthcare?
  - Prompt: Did you see you GP, cardiologist, go into hospital more/less often
- How would you change or improve the intervention to make it more helpful and engaging?
  - Prompt: more/less phone calls, more/less personalised or tailored content, other information content on website
